# New Insights into the Role of Matrix Metalloproteinases in Preeclampsia

**DOI:** 10.3390/ijms18071448

**Published:** 2017-07-20

**Authors:** Salvador Espino Y Sosa, Arturo Flores-Pliego, Aurora Espejel-Nuñez, Diana Medina-Bastidas, Felipe Vadillo-Ortega, Veronica Zaga-Clavellina, Guadalupe Estrada-Gutierrez

**Affiliations:** 1 Clinical Research Branch, Instituto Nacional de Perinatologia, Mexico City 11000, Mexico; salvadorespino@gmail.com; 2 Department of Immunobiochemistry, Instituto Nacional de Perinatologia, Mexico City 11000, Mexico; arturofpliego@gmail.com (A.F.-P.); auro.espejel@gmail.com (A.E.-N.); dianameedinab@gmail.com (D.M.-B.); v.zagaclavellina@gmail.com (V.Z.-C.); 3 Unidad de Vinculacion de la Facultad de Medicina, UNAM en el Instituto Nacional de Medicina Genomica, Mexico City 14610, Mexico; felipe.vadillo@gmail.com; 4 Research Division, Instituto Nacional de Perinatologia Isidro Espinosa de los Reyes, Mexico City 11000, Mexico

**Keywords:** preeclampsia, implantation, matrix metalloproteinases, trophoblast, endothelial damage, biomarkers, therapeutic targets

## Abstract

Preeclampsia is a severe pregnancy complication globally, characterized by poor placentation triggering vascular dysfunction. Matrix metalloproteinases (MMPs) exhibit proteolytic activity implicated in the efficiency of trophoblast invasion to the uterine wall, and a dysregulation of these enzymes has been linked to preeclampsia. A decrease in MMP-2 and MMP-9 interferes with the normal remodeling of spiral arteries at early pregnancy stages, leading to the initial pathophysiological changes observed in preeclampsia. Later in pregnancy, an elevation in MMP-2 and MMP-9 induces abnormal release of vasoactive factors conditioning hypertension. Although these two enzymes lead the scene, other MMPs like MMP-1 and MMP-14 seem to have a role in this pathology. This review gathers published recent evidence about the implications of different MMPs in preeclampsia, and the potential use of these enzymes as emergent biomarkers and biological therapeutic targets, focusing on studies involving human subjects.

## 1. Introduction

Matrix metalloproteinases (MMPs) comprise a family of 23 zinc and calcium-dependent proteases that degrade different components of the extracellular matrix. These enzymes are classified into collagenases, stromelysins, matrilysins, membrane-anchored MMPs, and others according to their specific substrate [1,2].

MMPs share common structural domains, an N-terminal propeptide region, a catalytic domain, a linker peptide (hinge region), and a C-terminal hemopexin like domain. The membrane-type MMPs (MT-MMPs) also contain an additional transmembrane domain, used to anchor the cellular surface [2,3,4]. In vivo, MMP activity is regulated mainly by the tissue inhibitors of MMPs (TIMPs 1–4), as well as transcriptional regulation, activation of the proenzyme state, and internalization by endocitosis [5,6,7,8].

MMPs have a wide tissue distribution and are responsible for degradation and turnover of extracellular matrix components in several physiological processes. Increased expression and activity of MMPs is linked to pathological conditions including cancer, chronic inflammation, as well as neurological and reproductive disorders [9,10,11,12]. Although the participation of several MMPs in reproductive pathologies such as preterm labor and premature rupture of membranes has been widely described [13,14,15], the role of these enzymes in preeclampsia represents an emerging area of interest for research.

Preeclampsia is a multisystem disorder of pregnancy defined by high blood pressure and proteinuria [16]. This pathology is a major perinatal problem in the western world, characterized by anti-angiogenesis, hypoxia, endothelial dysfunction, and immune modifications. Several studies suggest that the hallmark of preeclampsia is the impaired capacity of the trophoblast to invade the uterine spiral arteries, which results in a poorly perfused fetoplacental unit. This may lead to the secretion of factors into the maternal circulation, inducing endothelial dysfunction [17,18,19]. The identification of changes in the levels and activity of several MMPs as well as their endogenous inhibitors (TIMPs) in both defective trophoblast invasion and endothelial dysfunction led to the consideration of these proteases as key mediators in the pathological features of preeclampsia. This review describes recent research advances about the role of MMPs in early and late stages of preeclampsia, and the potential use of these enzymes as emergent biomarkers and biological therapeutic targets, focusing on studies involving human subjects.

## 2. MMPs in Trophoblast Implantation and Invasion in Normal Pregnancy and Preeclampsia

MMPs and their inhibitors play a major role in trophoblast invasion into the uterine wall. The profound changes in uterine microarchitecture required to transform the spiral vessels and create an optimum environment for embryonic development involve a grounding transformation in which MMPs are essential [20].

The blastocyst attachment to the uterine wall leads to a complex dialogue between membrane ligands and receptors to penetrate the epithelium and cross the basal lamina [21]. The trophoblastic cell layer differentiates in two types: the villous trophoblast that is responsible for fetal nutrition via expression of amino acid receptors, glucose, lipids, and oxygen, and the extravillous trophoblast, which binds to the uterine wall, transforming the vascular architecture. Some extravillous trophoblast cells from the anchorage villi change their phenotype and invade the endometrium by an interstitial route, expressing MMPs; they penetrate the inner myometrium until fusing to form multinucleate giant cells. Simultaneously, trophoblast cells migrate by an endovascular route until they reach the inner myometrium segments. At this point, trophoblast cells adhere to the vessel wall, secrete extracellular matrices, and form stellate protrusions. Probably both routes contribute to the transformation of the 120–140 spiral arteries that are necessary to supply the placenta.

Trophoblast cell invasion is precisely regulated by signaling events, autocrine and paracrine stimulus, specific protein recognition, and immunological tolerance [22]. This event is influenced by promoting (cytokines, growth factors, MMPs) and inhibiting factors (TIMPs). There are at least three cell lines in utero placental interface that express all MMPs with exception of MMP-20: trophoblast cells, endometrial stromal cells and natural killer cells. Decidual stromal cells in contact with trophoblast cells express very high levels of MMPs, optimizing their invasive potential.

The specific temporal characteristics of the trophoblast invasion lead to a differential expression of MMPs. During early stages of gestation, MMPs prepare the environment for the subsequent incursion to the placental bed. An elevated expression of pro-MMP-2 at 6–8 weeks dominates the scene over MMP-9 with subsequent declining concentrations, whereas pro-MMP-9 expression increases from 8 to 11 weeks, being the predominant gelatinase until the end of pregnancy [23], leading to the conclusion that MMP-2 has a major role during implantation and MMP-9 during invasion. A dysregulated secretion of these enzymes could interfere the physiological trophoblast invasion, i.e., the trophoblast in preeclampsia will produce less MMP-9 and MMP-9 inhibition or gene silencing, affecting trophoblast invasion in vitro [24]. Later in pregnancy, a downregulation of pro-MMP-3, and the active form of MMP-13 and -23, has been demonstrated, as well as an upregulation of pro-MMP-8, -14, -19 and -23 and the active forms of MMP-9, -10, -12, -14, -15, -16, -26, and -28 [25].

**Figure 1:**
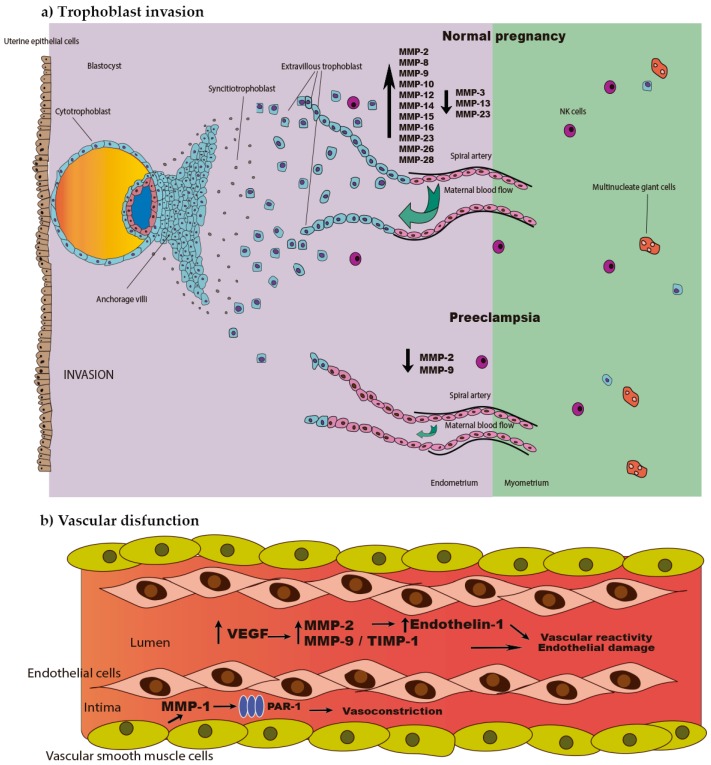
Role of matrix metalloproteinases (MMPs) in preeclampsia. (**a**) Impaired trophoblast invasion observed in early stage of preeclampsia is characterized by a decrease in MMP-2 and MMP-9, which affects spiral artery remodeling, causing a poorly perfused fetoplacental unit. (**b**) Vascular dysfunction developed during the late stage of preeclampsia is mediated by several MMPs including MMP-1, MMP-2 and MMP-9, which induce vasoconstriction, changes in vascular reactivity and endothelial damage. PAR-1, protease-activated receptor 1; VEGF, vascular endothelial growth factor; NK, natural killer cells; and TIMP-1, tissue inhibitor of matrix metalloproteinase-1.

Seval et al. described narrow ratios of MMP-2/TIMP-2 and MMP-9/TIMP-1 between 4 and 6 weeks of pregnancy, showing that there is a strict balance between MMPs and their inhibitors under physiological conditions [26]. Additionally, Rahat et al. demonstrated that this balance is regulated by DNA methylation as the promoter regions of these four enzymes are hypomethylated or completely unmethylated, allowing gene expression and the consequent trophoblast implantation and invasion. Increased MMP-2 and MMP-9 methylation is observed in villous samples of preeclamptic patients [27]; this gene silencing explains the low concentrations of these enzymes and supports the trophoblast invasion defects seen in preeclampsia (Figure 1a). Moreover, it has been reported that an elevated serum level of MMP-9 and a high ratio of MMP-2/TIMP-2 are associated with abortion [28].

Moreover, results of the studies carried out in late pregnancy are consistent with the findings described above for early pregnancy. Expression of different MMPs such as MMP-2, -8, -9, and -11 was downregulated in placental tissues from pregnancies complicated with preeclampsia at >35 weeks of gestation compared to normal pregnancies [29,30,31]. According to this, cultured cytotrophoblast cells purified from preeclamptic placentas are less invasive in vitro, expressing decreased levels of MMP-1, -7, -9, and -12 [32].

## 3. MMPs and Endothelial Dysfunction in Preeclampsia

There is plenty of evidence of the role of MMPs in placental dysfunction, and recently MMPs have become a target of interest in the vasculature of women with preeclampsia, due to their implication in vascular remodeling, angiogenesis and the uterine and systemic vasodilation during normal pregnancy [33]. Plasma levels of some MMPs and their inhibitors are altered in women with preeclampsia. It has been reported that MMP-2 plasma levels are elevated in preeclampsia [34], and that this event is mediated by the vascular endothelial growth factor (VEGF), which controls vascular permeability. Besides the effect on vascular remodeling, MMP-2 can mediate vascular reactivity by promoting the production of the vasoconstrictor peptide endothelin-1 through cleavage of the vasodilatory calcitonine gene related peptide [19,35]. Furthermore, MMP-2 elevation can be detected from the second trimester onwards in the plasma of women who subsequently develop preeclampsia.

Several studies show that MMP-9 increases along both, normal and preeclampsia-complicated pregnancy, while its inhibitor TIMP-1 increases in preeclamptic vs. normal pregnant women (Figure 1b) [19,36,37]. A significant increase of MMP-9 concentrations in serum from women who subsequently developed preeclampsia can be detected during the first and third trimester of gestation [38]. This evidence suggests that an imbalance between MMPs and their inhibitors could affect the vasculature of women with preeclampsia at the structural and functional levels, and that these changes can be detected even before the clinical symptoms appear.

Even though most evidence links increased circulating levels of MMP-9 and -2 with preeclampsia, two interesting reports show that pro-MMP-9, MMP-9 and MMP-9/TIMP-1 ratio remain unchanged in preeclamptic pregnancies in the Brazilian population [37,39]. These findings are supported by a report showing decreased MMP-9 plasma levels, although in this study there was no discrimination between pregnancy-induced hypertension with or without proteinuria [40].

Plasma of women with preeclampsia and, interestingly, plasma of non-pregnant women, significantly enhanced the myogenic tone and blunted relaxation of mesenteric arteries from virgin female C57BL/6J mice. This model was also used to evaluate the effect of the plasma when exposed along with MMP inhibitors and results showed that myogenic tone increased and the relaxation was abrogated only in vessels incubated with plasma from patients with preeclampsia, and not of those incubated with plasma from non-pregnant women. This finding is contradictory to the premise that MMPs cause systemic vasoconstriction in preeclampsia [18].

We evaluated the role of MMP-1 in preeclampsia, showing a high level of this MMP in the vasculature of women with this pathology, suggesting a role in the vascular collagen breakdown that possibly favors the edema and proteinuria observed in these patients. In this work we also demonstrated that MMP-1 secreted by vascular smooth muscle cells induces the release of interleukin-8, which favors the recruitment of activated neutrophils in women with preeclampsia, and the consequent generation of reactive oxygen species. Additionally, vascular reactivity mediated by MMP-1 was tested using intact omental arteries in the presence of a potent and selective protease-activated receptor-1 (PAR-1) antagonist, and we found that this MMP has potent vasoconstriction properties that are PAR-1 dependent [41]. Recent findings suggest that MMP-1 enhances vascular reactivity to vasoconstrictor hormones such as angiotensin II, which are mediated by an endothelial PAR-1, ras homolog family member A (RhoA) kinase, and endothelin-1 pathway [42].

We have also explored the possible contribution of DNA methylation to the altered expression of genes involved in collagen metabolism using omental arteries from normal pregnant and preeclamptic women. We found that many genes from the MMP family have different methylation patterns among the study groups; *MMP-1*, *-8*, *-12*, *-13*, *-21* and *-26* were hypomethylated in the promoter region in the samples from women with preeclampsia. We further evaluated the effect of hypomethylation of *MMP-1* in cultured vascular smooth muscle cells stimulated with neutrophils as an in vitro model of preeclampsia showing a strong correlation with the increase of *MMP-1* gene expression [43,44]. These findings suggest the possibility that epigenetic mechanisms directly involving the promoters of the target collagen metabolism genes, or possibly genes that regulate their expression (e.g., transcription factors), play an important role in the vascular dysfunction associated with preeclampsia.

## 4. MMPs as New Biomarkers and Potential Biological Targets in Preeclampsia

The identification of accurate, sensitive and specific biomarkers in preeclampsia is crucial for diagnosis and prognosis of this syndrome at early pregnancy stages. MMPs are implicated in a number of key pathophysiological processes representing potential therapeutic and diagnostic targets. As a result of research at different levels, such as gene expression, protein concentration and enzymatic activity in distinct biological samples, MMPs have been postulated as likely biomarkers for preeclampsia [45].

MMP-2 and MMP-9 have become of particular interest due to their frequent implications as key factors in the pathogenesis of preeclampsia. Feng et al. investigated the reliability of these two proteases, and their relative ratio in plasma, to predict preeclampsia. Plasma concentration of MMP-2 and MMP-9 at 20 weeks of gestation was measured in women with suspected preeclampsia. The study showed that the ratio of MMP-2/MMP-9 was significantly elevated in preeclamptic women, with high specificity and sensitivity, thus distinguishing pregnancies complicated with preeclampsia from healthy pregnancies, resulting in an accurate biomarker in a high-risk population during the second trimester of gestation [46]. Additionally, the potential value of maternal serum MMP-9 in first-trimester screening for preeclampsia was tested, showing no improvement in the predictive value of the model so far [47].

Urine samples from healthy and preeclamptic pregnancies were analyzed in a study that was carried out in order to predict the risk of developing preeclampsia at three different stages of early pregnancy (12, 16 and 20 gestational week). From a set of nine MMPs evaluated, only MMP-2 was found to be significantly higher at 12 and 16 weeks [48]. In a different study, 14 biomarkers for preeclampsia, including MMP-2 and MMP-9, were evaluated in urine samples. At delivery, urine concentrations of MMP-2 and MMP-9 were significantly elevated in women with severe preeclampsia compared to normal pregnancy, and this feature persisted 6 to 8 weeks after delivery [49].

The reported association between *MMP* gene polymorphisms and preeclampsia remains controversial, showing inconclusive or inconsistent results. An early report regarding MMP-9-1562 C/T polymorphism showed that women carrying the T allele were less likely to develop preeclampsia [50]. Contrary to this finding, the same variant of this MMP-9 polymorphism has been associated with a higher risk of gestational hypertension and preeclampsia in Kurdish and Chinese population [51,52]; also, some results suggest that this polymorphism may affect the therapeutic response to antihypertensive agents, concluding that this MMP-9 variant could help to identify those patients with a refractory response [37]. Additionally, Luizon et al. reported that the combination of genotypes MMP-9-1562CC with VEGF-634CC or MMP-9-1562CT with VEGF-634CC or -634GG were significantly more frequent in women with preeclampsia than in normal pregnant women, and results suggest that these epistasis contribute to a higher susceptibility to developing preeclampsia [53]. Different results were reported in Brazilian and British women, where the MMP-9-1562 C/T polymorphism was not associated with the syndrome [54,55]. Furthermore, two meta-analysis involving different studies of this MMP-9 polymorphism showed that the genetic variants were not associated with development of preeclampsia [56,57]. The controversial findings may be due to all of these studies being performed in different ethnic groups. On the other hand, MMP-2 polymorphisms (g.–1306C>T and g.–735C>T) do not seem to be associated with hypertensive disorders during pregnancy nor pharmacological response [34,55,58].

MMP-14 has been proposed as a potential therapeutic target to reduce circulating soluble endoglin (sEng) and mitigate clinical manifestations of preeclampsia. The link between a poor placentation and endothelial dysfunction can be understood by the release of two anti-angiogenic factors: sEng and soluble fms-like tyrosine kinase-1 (sFlt-1). Kaitu’u-Lino et al. reported that MMP-14 cleavages placental endoglin to release the sEng form to peripheral circulation, which antagonizes transforming growth factor-β (TGF-β), contributing to the endothelial dysfunction observed in preeclampsia. Moreover, MMP-14 and -15 have been evidenced in syncitiotrophoblasts, and are known to be downregulated by endothelin-1 during first trimester inhibiting trophoblastic migration and invasion [59,60,61,62].

As mentioned before, the elevation of MMP-1 and MMP-2 is strongly associated with the endothelial dysfunction observed in preeclamptic women [41,42,63,64]. Due to the fact that these two MMPs can be found in maternal circulation, the control of their activity represents an ideal therapeutic strategy for blocking the molecular mechanisms that lead to hypertension in preeclampsia. Natural and synthetic MMP inhibitors are considered as therapeutic strategies to control MMPs proteolytic activity in several pathological models, mainly cardiovascular disease. Since these molecules cause adverse side effects, further research needs to be conducted to consider them as reliable therapeutic options in preeclampsia and other pathologies [65,66].

Finally, novel epigenetic studies suggest the involvement of miRNAs in preeclampsia and other gestational pathologies. In silico and in vitro analysis have identified several miRNA-mRNA regulatory mechanisms that may contribute to the pathogenesis of preeclampsia [67,68]. Mayor-Lynn et al. provided evidence that preeclamptic placentas show an altered expression of several miRNAs with potential regulatory functions on the expression of MMP-1, MMP-9 and TIMP-3 [69]. A recent work demonstrated that miR-93 is elevated in preeclampsia and inhibits MMP-2, reducing migration and invasion of trophoblast cells [70]. miR-346 and miR-582-3-p were evaluated in vitro to determine their regulatory effect on trophoblast biology. Results showed that these miRNAs downregulate endocrine gland-derived endothelial growth factor (EG-VEGF), inhibiting MMP-2 and -9 expression and activity as well as trophoblast migration [71]. High plasma levels of miR-855-5p observed in preeclampsia and the negative correlation with MMP-9 protein plasma levels open new insights to explore this miRNA as a valuable therapeutic option [72]. Although miRNAs may represent a cutting edge therapeutic target for preeclampsia intervention, further clinical studies are required to validate their role in this pathology.

In conclusion, several studies show that at the initial stage of pregnancy, a low concentration of placental MMPs may affect the spiral artery remodeling, causing a poorly perfused feto-placental unit. Vascular dysfunction observed during the late stage of preeclampsia could be mediated by several MMPs inducing vasoconstriction, changes in vascular reactivity and endothelial damage. For these reasons, MMPs have become striking biomarkers to identify women with a high risk of developing preeclampsia, as well as eligible biological targets for treating women with this syndrome. However, it is important to pursue larger basic and clinical studies, as well as meta-analysis to evaluate whether MMPs play a decisive role in the pathophysiology of preeclampsia and the predictive value of these enzymes as biomarkers or therapeutic targets.
